# Information quality assessment and content analysis of dementia prevention on WeChat: a cross-sectional study

**DOI:** 10.3389/fpubh.2025.1666853

**Published:** 2025-11-19

**Authors:** Yashi Lin, Zhouxin Li, Kairou Zhang, Gang Liu, Ruiyu Huang, Yanxia Guo, Xiaofang Yang, Baolu Zhang

**Affiliations:** 1 Department of Nursing, The Affiliated Hospital, Southwest Medical University, Luzhou, China; 2 School of Nursing, Southwest Medical University, Luzhou, China; 3 Department of Orthopedics and Center for Orthopedic Diseases Research, Affiliated Traditional Chinese Medicine Hospital of Southwest Medical University, Luzhou, China; 4 School of Continuing Education, Guiyang Healthcare Vocational University, Guiyang, China; 5 Faculty of Nursing and Midwifery, Jiangsu College of Nursing, Huai'an, China; 6 Guiyang Maternal and Child Health Care Hospital, Guiyang, China

**Keywords:** dementia prevention, WeChat, information quality, content analysis, health information

## Abstract

**Objective:**

This study aimed to evaluate the information quality and content of dementia prevention on WeChat.

**Methods:**

The search term “dementia prevention” was used on WeChat, resulting in 125 samples being included. Information quality was assessed using GQS and PEMAT-P. The content was evaluated based on dementia prevention guidelines and article characteristics.

**Results:**

Information quality was moderate (median 3.0), with high understandability and actionability. Most articles were published by medical institutions (37.6%), but governmental organizations achieved the highest scores (*p* < 0.05). Content completeness was low, with healthy lifestyle being mentioned most frequently (98.4%), while sensory organ protection and improving air environment were mentioned least frequently (both at 3.2%). Articles with more complete content and fewer advertisements demonstrated significantly higher information quality (*p* < 0.001 and *p* = 0.016, respectively).

**Conclusion:**

Overall, the information quality of dementia prevention on WeChat was medium, with high understandability and actionability but low content completeness. Articles with more complete content and fewer advertisements have better information quality. It is recommended that publishers provide more complete articles, while platforms should strengthen advertisement supervision.

## 1 Introduction

Dementia is an acquired loss of cognition in multiple cognitive domains sufficiently severe to affect social or occupational function (1). Dementia currently affects an estimated 50 million people worldwide (2), with projections indicating that this number could rise to 152 million by the middle of the century (3). China has the largest population of people with dementia in the world (4). Dementia places a significant burden on patients’ families and healthcare systems, although there are drugs that can slow disease progression or address symptoms, prevention remains critically important, given the limited curative options available (5, 6). Taking action now on dementia prevention will greatly improve the quality of life for patients and their families (7).

With the rapid advancement of information technology, the Internet has become the primary source of medical information for the public and patients (8). WeChat has emerged as the most widely used social platform in China (9). In 2023 alone, over 448 million articles were published on WeChat (10). Surveys have shown that 98.35% of respondents have accessed health information via WeChat, with 97.68% engaging with such content. Additionally, 32.33% of respondents reported regularly reading health education articles on WeChat (9). These findings underscore WeChat’s dominant position in China, serving as both the most widely used platform among Chinese users and their primary source of health information.

Researchers have evaluated the quality of dementia-related information across various digital platforms. Traditional social media platforms have shown mixed results, with TikTok videos about dementia demonstrating poor information quality (11), while YouTube content on dementia-related topics has shown higher information quality (12). The emergence of generative artificial intelligence tools has introduced new dynamics to health information seeking. Hristidis et al. compared ChatGPT with Google search results for dementia-related queries, finding that while ChatGPT provided more objective responses, it lacked source attribution and currency compared to traditional search engines (13). Additionally, Aguirre et al. found that ChatGPT provided high-quality responses to dementia caregivers’ questions, with particular strengths in synthesizing information and providing recommendations, though with limitations in completeness (14).

Notably, existing studies have primarily focused on general dementia information or comprehensive content (11, 12), while assessments of information quality specifically targeting the critical area of dementia prevention remain a research gap. As dementia prevention represents the most cost-effective and actionable intervention strategy available, the accuracy and reliability of related information hold particular importance for public health (7). Current researchers have evaluated the information quality of health-related articles on WeChat, such as hypertension and diabetes-related articles showing lower information quality (15, 16), and breast cancer treatment-related articles demonstrating moderate information quality (17), but content analysis specifically focusing on dementia prevention as a distinct topic on WeChat has not been conducted. Therefore, this study pioneered the specific evaluation of information quality and content of dementia prevention-related articles on WeChat.

## 2 Materials and methods

### 2.1 Search strategy and data collection

Our study was conducted on November 6, 2024, using the keyword “痴呆预防” (which means “dementia prevention”) in the WeChat search bar. We selected this term because “痴呆” has been continuously used from traditional Chinese medicine through contemporary biomedical practice, making it the most representative term for how the general Chinese population conceptualizes this condition (18). To verify the potential impact of different search terms, we conducted an additional validation analysis on August 30, 2025, using “失智预防” as a supplementary search term, and the main findings were consistent with the original study, as detailed in Supplementary Tables 1–2. Given WeChat’s dynamic content ecosystem, where articles are continuously updated, published, and removed while search algorithms undergo daily modifications, we adopted a cross-sectional snapshot design to ensure methodological consistency and data reliability. All searches were completed within a single day with the purpose of “maintaining sample consistency,” that is, ensuring all articles were retrieved under identical search algorithm conditions, avoiding systematic bias that could arise from the WeChat platform’s daily content updates and algorithm adjustments. Snapshot analysis is a research methodology that captures and analyzes data at one specific time point, eliminating temporal variations and ensuring all retrieved content is evaluated under identical conditions. This approach has been widely employed in health information quality research across various social media platforms, including studies evaluating YouTube video content quality (19). The search was performed in the “Articles” section. The search was performed in the “Articles” section, which is specifically designed to retrieve text content, excluding other media formats such as videos. WeChat offers three primary sorting options for search results: “All,” “Latest,” and “Most Popular.” To minimize the impact of external factors on the search results, the “All” sorting option was selected, which is the default setting used by the general public. We conducted an exhaustive search by reviewing all search result pages until no additional content appeared, and saved all 196 retrieved article links in Microsoft Excel.

**Figure 1:**
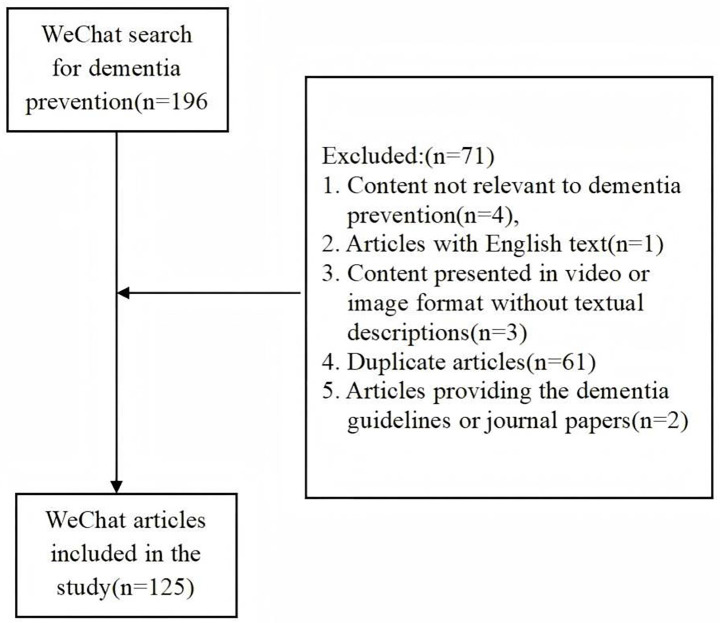
Dementia prevention article screening flowchart on the WeChat platform.

Additionally, to address concerns about potential limitations of single-time-point data collection, we conducted a complete replication study 9 months after the original research (August 28, 2025). The validation results showed that the main research findings were statistically highly consistent with the original results, indicating the temporal stability of dementia prevention information quality patterns on the WeChat platform. This finding supports the validity of our snapshot analysis approach and demonstrates that data collected within a single day has good representativeness, as detailed in Supplementary Tables 3–4. Exclusion criteria included (1) content not relevant to dementia prevention, (2) articles with English text, (3) content presented in video or image format without textual descriptions, (4) duplicate articles, and (5) articles providing the dementia guidelines or journal papers. After the screening, 125 articles were retained for further data extraction and analysis (Figure 1). Although the final sample size was 125 articles, this number reflects the true state of WeChat’s content ecosystem. Unlike Twitter’s character limitations and Facebook’s brief posts (20, 21), WeChat articles feature long-form, in-depth characteristics with higher information density than other social media short-form content. Meanwhile, among the initially retrieved 196 articles, a large portion consisted of duplicate reposts, which is a typical characteristic of the WeChat platform. Through rigorous deduplication and relevance screening, the 125 articles represent all unique dementia prevention content available at the study time point, ensuring sample completeness and representativeness.

The screening process was conducted collaboratively by researchers A and B. In the first stage, they jointly excluded articles unrelated to dementia prevention and those published entirely in English by examining titles and quickly reviewing content. Then, researchers A and B manually assessed and excluded articles containing videos or images without textual descriptions. The study team then recorded the text content of each article in Excel and identified duplicate articles through text comparison, retaining the earliest published version. In the final stage, researchers A and B carefully reviewed all remaining articles and excluded those directly providing dementia guidelines or journal papers, as these highly specialized medical materials are difficult for ordinary WeChat users to understand and often fail to serve an educational purpose for the general public.

Researcher A completed the data extraction from the articles. Researcher A extracted information, including basic article characteristics (such as title, number of likes, number of views, number of shares, whether references were cited, and whether advertisements were present) as well as relevant information collected from the public account’s homepage (such as account name, certifying entity, and certification type). All numbers of views were obtained exclusively from the WeChat platform. Since each device only registers one view per article regardless of multiple accesses, the reported view counts accurately reflect user engagement without inflation from our screening process. Additionally, researchers did not like or share any of the articles during the data collection process. All extracted data were systematically recorded in Microsoft Excel. Based on the collected certifying entities and certification types, researchers A and B, after discussion, classified article publishers into five categories. These categories included governmental organizations, commercial organizations, medical institutions, media or social organizations, and individuals. When they encountered disagreements during the classification process, they consulted with researcher C and reached a consensus through discussion.

### 2.2 Evaluation methodology and procedure

Our study assessed WeChat articles on dementia prevention from information quality and content. To evaluate information quality, our study chose to apply the Global Quality Scale (GQS) and the Patient Education Materials Assessment Tool for Printable Materials (PEMAT-P). To ensure the scientific rigor and accuracy of the research, we invited two psychiatrists with extensive clinical experience to independently complete the information quality assessment. GQS is a five-point scale assessment tool specifically designed for the overall evaluation of information quality, with a particular focus on information fluency and usability (22). It is widely applied in various fields for assessing health information quality (23, 24). According to the GQS scoring criteria, information scoring 4–5 points is categorized as high quality, 3 points as medium quality, and 1–2 points as low quality (22). Detailed scoring criteria are provided in Supplementary Table 5.

PEMAT-P is primarily used to evaluate patient-oriented educational printable materials, focusing on their understandability and actionability, helping to determine whether health information is easy to understand and implement. This assessment tool has been widely applied in evaluating paper or printable health education materials (25). PEMAT-P includes 24 items, with 17 items assessing understandability and 7 items assessing actionability (26). Each item is scored using “agree” (1 point), “disagree” (0 points), or “not applicable” (NA). The final score is determined by calculating the percentage of “agree” responses among all applicable items, excluding items rated as “not applicable.” A PEMAT-P score exceeding 70% indicates that the material has a high level of understandability and actionability. In comparison, a score below 70% suggests that the material may lack sufficient clarity or practicality (27). Complete scoring criteria are presented in Supplementary Table 6. Using IBM SPSS Statistics 29.0, we calculated the inter-rater reliability between the two raters. The results showed an inter-rater reliability coefficient of 0.826 for the GQS tool and 0.832 for the PEMAT-P tool, indicating satisfactory consistency levels between raters.

The same two psychiatrists also independently analyzed dementia prevention content in the articles. We categorized dementia prevention content into 9 aspects. These aspects include (1) Education, (2) Sensory organ protection, (3) Chronic disease management, (4) Healthy lifestyle, (5) Social interaction, (6) Avoiding brain trauma, (7) Mental health management, (8) Improving air environment, and (9) Traditional Chinese medicine (TCM) prevention. Our evaluation was primarily based on the Dementia Prevention, Intervention, and Care: 2024 report of the Lancet Standing Commission (28). After researchers found TCM prevention content in the articles, we incorporated TCM-based dementia prevention literature (29–31), which enriched our analysis and provided a broader perspective on prevention strategies. Each aspect was operationalized as a dichotomous variable, categorized as ‘mentioned’ or ‘not mentioned’ based on explicit coding criteria. Articles were coded as ‘mentioned’ (1) if they contained at least one complete sentence providing specific information, recommendations, or actionable advice related to the prevention strategy. Generic mentions without substantive content were coded as ‘not mentioned’ (0). The detailed operational definitions and coding criteria for all nine categories are provided in Supplementary Table 7. To ensure coding reproducibility and objectivity, both psychiatrists underwent standardized training using the operational definitions outlined in Supplementary Table 7. The coding procedure was conducted in two phases: first, they independently coded a pilot sample of 20 articles to establish baseline agreement and refine ambiguous coding decisions through consensus discussion. Subsequently, they independently coded the remaining 105 articles. The psychiatrists evaluated dementia prevention content from two aspects: the percentage of each prevention topic mentioned across all articles and the completeness score of individual articles. For content completeness scoring, each prevention topic received a score of ‘1’ if mentioned according to the operational criteria and ‘0’ otherwise, with the total score representing the article’s completeness level. Based on the content evaluation of all 125 articles, the inter-rater reliability coefficient was 0.814, indicating good agreement between the two raters.

After the two clinicians completed all information quality and content assessments, we conducted a systematic comparison of the two raters’ scores to identify scoring discrepancies. After a comparative analysis, disagreements were found in 38 articles (30.4% of the total sample) between the two psychiatrists. These disagreements were primarily concentrated in content analysis (27 articles, 21.6%), particularly in identifying sensory organ protection content (18 articles, 14.4%). Additionally, disagreements occurred in GQS scoring (15 articles, 12.0%) and PEMAT-P scoring (18 articles, 14.4%). All disagreements were systematically resolved through consultation with a psychiatrist specializing in dementia research, achieving a final consensus. It should be noted that some articles had disagreements across multiple assessment dimensions; therefore, the sum of individual disagreement categories exceeds the total number of 38 articles with disagreements.

### 2.3 Statistical analysis

Our study conducted data analysis using IBM SPSS Statistics 29.0. Variables were classified into categorical and continuous variables. Categorical variables were described using frequencies (%), while continuous variables were presented as medians (interquartile range, IQR) due to their non-normal distribution. The Kruskal-Wallis H test was used to compare whether there were differences in GQS scores, PEMAT-P scores, and content completeness scores among different publishers. Correlational analyses are widely used in health information quality research. Studies on hypertension information quality on WeChat and diabetes information quality have employed such analyses to identify key factors affecting information quality and their interactions (15, 16). Therefore, our study also adopted correlational analyses to examine these relationships. Spearman correlation analysis evaluated the relationships between GQS scores, PEMAT-P scores, Content completeness scores, number of likes, and number of views; *p* < 0.05 was considered statistically significant.

### 2.4 Ethical considerations

This study involved the analysis of publicly available articles on the WeChat platform. The study focused on publicly accessible content rather than recruiting human participants directly. We have obtained an Ethics Review Exemption Statement from the ethics committee of the institution where this research was conducted, confirming that this type of study, analyzing only publicly available information, does not require ethical review. Our study methodology adheres to WeChat Public Platform regulations, does not collect personal privacy information, and protects privacy by de-identifying all data during analysis and presenting findings only in aggregate form.

## 3 Results

### 3.1 Characteristics of the articles

Table 1 shows the basic characteristics of dementia prevention articles on WeChat. Among them, 11.2% of the articles cited references. Regarding advertising, 10.4% of the articles contained advertisements. Most articles were published by medical institutions (37.6%). In terms of views, the median was 1325.0 (IQR 4846.0). For the number of likes, the median was 10.0 (IQR 64.0). As for the number of shares, the median was 4.0 (IQR 27.0).

**Table 1:** Characteristics of articles on dementia prevention on WeChat (*n* = 125).

Variable	Statistics
Reference source, *n* (%)	
Yes	14 (11.2%)
No	111 (88.8%)
Advertising, *n* (%)	
Yes	13 (10.4%)
No	112 (89.6%)
Article publishers, *n* (%)	
Governmental organizations	14 (11.2%)
Commercial organizations	31 (24.8%)
Medical institutions	47 (37.6%)
Media or social organizations	10 (8%)
Individuals	23 (18.4%)
Number of views, median (IQR)	1325.0 (4846.0)
Number of likes, median (IQR)	10.0 (64.0)
Number of shares, median (IQR)	4.0 (27.0)

### 3.2 Information quality

We evaluated the overall information quality, understandability, and actionability of dementia prevention articles on WeChat, categorizing them by different publishers. Regarding overall information quality, the articles’ GQS scores were medium (median 3.0). In terms of understandability, the articles’ overall scores were relatively high (median 88.0%). For actionability, the articles also achieved high scores (median 80.0%). We compared information quality metrics across the five publisher categories using Kruskal–Wallis H tests. There were significant differences among publishers in understandability (*p* = 0.006) and actionability (*p* = 0.007). Government organizations produced articles with the highest scores, while individual publishers had the lowest understandability (median 79.5%) (Table 2).

**Table 2:** The difference analysis of different publishers in GQS scores, PEMAT-P scores, and content completeness.

Variable median (IQR)	Overall (*n* = 125)	Governmental organizations	Commercial organizations	Medical institutions	Media or social organizations	Individuals	*p*-value
GQS^a^	3.0 (1.0)	4.0 (2.0)	3.0 (1.0)	3.0 (1.0)	3.0 (1.0)	3.0 (1.0)	0.201
PEMAT-P^a^							
Understandability (%)	88.0 (13.0)	94.0 (6.0)	88.0 (13.0)	88.0 (7.0)	84.5 (13.0)	79.5 (18.0)	0.006^*^
Actionability (%)	80.0 (0.0)	83.0 (20.0)	80.0 (0.0)	80.0 (20.0)	80.0 (20.0)	80.0 (20.0)	0.007^*^
Content completeness^a^	4.0 (2.0)	5.0 (2.0)	4.0 (2.0)	5.0 (2.0)	3.0 (3.0)	4.0 (2.0)	0.165

a Kruskal–Wallis *H* test comparing differences among different publishers.

* *p* < 0.05.

### 3.3 Content analysis

**Figure 2:**
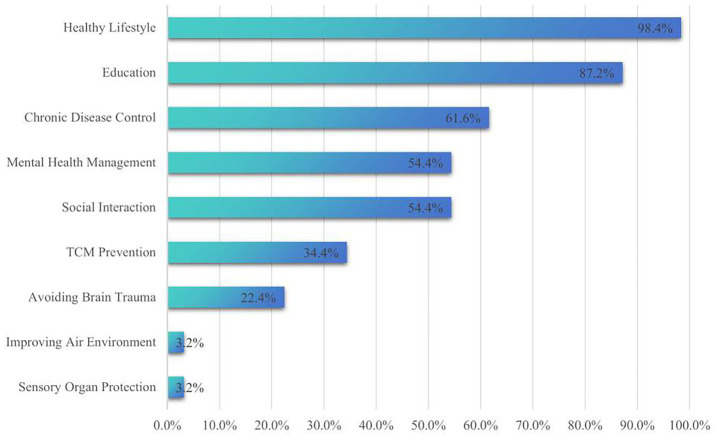
The percentage of each dementia prevention content.

In terms of content categories related to dementia prevention, the most frequently mentioned topics were healthy lifestyle (98.4%), while the least mentioned were sensory organ protection (3.2%) and improving air environment (3.2%) (Figure 2). The completeness of the dementia prevention information was assessed on a scale from 1 to 9, with an overall median score of 4 (IQR 2) (Table 2).

### 3.4 Correlation analysis

The correlation analysis shows a significant correlation between GQS and content completeness scores (*p* < 0.001). Additionally, the presence of advertisements in articles was significantly correlated with GQS scores (*p* = 0.016) and actionability (*p* = 0.016). The actionability score of PEMAT-P was correlated with content completeness (*p* = 0.016) (Table 3).

**Table 3:** Correlation of PEMAT-P score, content completeness, number of views, and number of likes.

Variable	GQS		PEMAT-P			
			Understandability		Actionability	
	*r*-value	*p*-value	*r*-value	*p*-value	*r*-value	*p-*value
Content completeness^a^	0.832	<0.001^**^	0.036	0.689	0.216	0.016^*^
Number of views^a^	−0.030	0.743	−0.068	0.451	0.018	0.840
Number of Likes^a^	−0.034	0.708	−0.041	0.647	−0.003	0.973
Advertising^a^	−0.216	0.016^*^	−0.163	0.069	−0.216	0.016^*^

a Spearman correlation analysis.

* *p* < 0.05.

** *p* < 0.001.

## 4 Discussion

### 4.1 Principal findings

Our study evaluated the information quality and content of dementia prevention-related articles on the WeChat platform. Regarding information quality, the overall quality was moderate, while understandability and actionability were relatively high. Medical institutions were the main publishers of dementia prevention articles, but articles published by government organizations demonstrated the best performance in understandability and actionability. In terms of content, overall completeness was less than ideal, with healthy lifestyle being the most frequently mentioned, while sensory organ protection and improving air environment were mentioned the least. Articles with more complete preventive content and fewer advertisements demonstrated higher information quality and actionability.

### 4.2 Information quality and article publishers

Our research shows that the overall information quality of dementia prevention articles is at a moderate level. This moderate quality level places WeChat within a consistent pattern observed internationally across different social media platforms and health topics, suggesting that moderate-quality health information may be a characteristic feature of health communication on social media. For example, health information about amputation rehabilitation and meniscus tear rehabilitation on social media platforms follows this pattern (32, 33) but differs from studies on hypertension treatment on WeChat and asthma-related content on Twitter (15, 34). These differences may be attributed to the use of different assessment tools—DISCERN primarily evaluates the quality of treatment-related information (35), while GQS provides a broader assessment of overall information quality (36). Furthermore, our study found that medical institutions are the primary publishers of dementia prevention-related content on the WeChat platform, which is consistent with research on online educational videos about pre-dialysis chronic kidney disease (37). This pattern reflects the growing recognition among professional medical organizations worldwide of their responsibility to provide health education through social media channels. At the same time, disease health education requires deep medical expertise, making it difficult for non-professionals to accurately understand and disseminate related information. Therefore, we recommend establishing unified digital health information quality standards, creating certification mechanisms for medical institutions publishing health information, and providing professional training and guidance for non-professional content creators. Simultaneously, we should establish a health information quality assessment system to ensure that health information on digital platforms meets quality standards.

Our study indicates that dementia prevention articles on WeChat demonstrate high understandability, which aligns with findings from studies on breast cancer survivors and type 2 diabetes (38, 39). However, Hristidis et al. compared ChatGPT with Google search results for dementia-related queries, finding that while ChatGPT provided more objective responses with higher relevance scores, both platforms demonstrated poor readability (13). Additionally, Dosso et al. found that ChatGPT responses about dementia averaged a 12–13th-grade reading level, significantly higher than recommended health literacy standards (40). In contrast, our WeChat articles achieved high understandability scores, suggesting that traditional text-based social media platforms may offer superior accessibility compared to AI-generated content or search engine results. Similarly, these WeChat articles exhibit strong actionability, consistent with a study on patient education materials for sepsis (41), but differ from study findings on adolescent vision health information on TikTok and chronic kidney disease information on YouTube (37, 42). This variation in information quality across platforms reflects fundamental differences in platform architecture and content delivery mechanisms. WeChat, as a text-based platform with unique characteristics of long-form content supplemented by images (43), may offer different patterns of information accessibility compared to AI-generated responses; and compared to the time-constrained formats of TikTok and YouTube, which primarily rely on short videos, it is easier to provide understandable, actionable guidance (44). This platform-differentiated approach requires public health professionals to possess sophisticated digital health literacy, while public health agencies should recognize that platform-specific characteristics influence health information effectiveness and adjust their communication strategies accordingly by developing platform-specific content creation guidelines.

Our study found that dementia prevention articles published by government organizations tend to have higher understandability and actionability, a finding consistent with pre-dialysis chronic kidney disease research (37). This may be attributed to the government’s potential role in providing authoritative information and establishing clear standards while offering reliable official health information resources (45). Compared with international trends, the digital health initiatives of the US National Institute on Aging (21) and the UK NHS dementia strategy (46) all emphasize the important role of authoritative government agencies in health information dissemination, which is consistent with our finding that government agencies publish higher quality content. This indicates that global public health agencies have a unique opportunity to leverage official social media channels for dementia prevention education, rather than primarily relying on private entities to fill information gaps. In contrast, dementia prevention articles published by individual users often have lower understandability. This may be due to individual content creators’ lack of medical or health communication expertise, making it difficult for them to communicate complex medical terminology effectively. We recommend that individuals should actively participate in relevant health communication training to improve their medical literacy, while also utilizing appropriate charts and illustrations to supplement written explanations to enhance article understandability. Public health agencies should also develop corresponding policies to strengthen the assessment and supervision of articles published by individuals.

### 4.3 Dementia prevention content

Regarding article content, our study found that the content completeness of the articles was relatively low. This is consistent with the findings from studies on diabetes-related article content completeness (47), indicating that the completeness of health-related content on social media platforms remains a significant challenge globally. Even though completeness prevention guidelines exist internationally, their translation into public-facing digital content remains incomplete across different cultural and platform contexts. Healthy lifestyle is the most frequently mentioned preventive content, while sensory organ protection and improving air environment are mentioned the least. However, relevant research indicates that protecting sensory organs and improving air environment can reduce the risk of dementia (48–50), representing prevention strategies with significant potential. We recommend that article publishers pay more attention to content on sensory organ protection and improving air environment when conducting health education on dementia prevention. This content can include protective measures for sensory organs such as hearing and vision, as well as solutions for optimizing indoor air quality. Meanwhile, public health agencies should develop content frameworks to ensure complete coverage of all scientifically supported prevention strategies. Given the global and widespread nature of health information quality issues, this study’s evaluation methods and findings may serve as a reference for relevant international organizations in developing digital health information quality standards, thereby contributing to improved health communication effectiveness in digital environments.

### 4.4 Correlation between content completeness, advertisements, and information quality

Our study indicates that more complete articles demonstrate higher information quality and actionability. This is consistent with the results of the study on hypertension on WeChat (15), because complete articles provide a broader understanding of preventive measures and simultaneously require more systematic professional explanations and scientific communication, thereby enhancing their overall information quality and actionability. Notably, our study found that dementia prevention articles containing advertisements tend to be lower quality and less actionable. This may be attributed to the fact that most ad-containing articles are published by commercial organizations, whose content may be driven by economic interests rather than scientific rigor (51, 52), potentially leading to misleading or less reliable information. In light of these findings, we recommend that publishers ensure complete coverage of preventive content when disseminating dementia prevention information, and that platforms implement stricter oversight, quality review, and transparency labeling mechanisms for health-related articles containing advertisements to improve information quality and reduce the negative impact of commercial interests on scientific accuracy.

### 4.5 Limitations and future directions

First, we used only one search term (“痴呆预防”) to identify relevant articles, which may have resulted in missing content that uses alternative terminology such as “失智预防” (cognitive impairment prevention), “认知障碍预防” (cognitive disorder prevention), or “阿尔茨海默病预防” (Alzheimer’s disease prevention). Future studies should employ multiple search terms and synonyms to capture a more complete picture of dementia prevention information on social media platforms. Second, this study represents a temporal snapshot of WeChat content captured on a single date, which may not reflect the dynamic nature of social media information over time. Future studies should consider adopting longitudinal research designs, collecting data at multiple time points to observe temporal trends in the quality of dementia prevention information. Third, we primarily evaluated the information quality related to dementia prevention, possibly overlooking other aspects of dementia. We recommend that future studies cover multiple aspects of dementia, including diagnosis, treatment, and care. Additionally, while our study captured all available dementia prevention articles through WeChat’s search function on the study date, it still lacks sufficient sample size. Future research should employ multiple search terms and synonyms to capture a more comprehensive range of dementia prevention content. Finally, this study mainly analyzed the information quality on the WeChat platform, potentially overlooking the information quality regarding dementia prevention on other platforms. We suggest that future studies could analyze the information quality on dementia prevention across other platforms.

## 5 Conclusion

Our study evaluated the information quality and content of dementia prevention materials on the WeChat platform. The findings revealed that the overall information quality was at a medium level, with relatively high understandability and actionability of articles, especially those published by government organizations. However, content completeness remains less than ideal, with minimal mention of sensory organ protection and improving air environment. Articles with more complete content and fewer advertisements have significantly better information quality. We suggest that publishers should create better, more complete articles about preventing dementia. They should especially talk more about protecting your senses and improving air quality. The people who regulate WeChat should create tougher rules and closely monitor advertisements.

## Data Availability

The raw data supporting the conclusions of this article will be made available by the authors, without undue reservation.
