# Supplementary material for: Information quality assessment and content analysis of dementia prevention on WeChat: a cross-sectional study
Source: Front Public Health. 2025 Nov 19;13:1666853. doi: 10.3389/fpubh.2025.1666853 (PMC12672899; doi:10.3389/fpubh.2025.1666853)
Supplement: Supplementary file 1 [file Supplementary_file_1.docx]

Supplementary Material

**Supplementary Table 1**: The difference analysis of different sources in GQS scores, PEMAT-P scores and content completeness. (Results from searches conducted using the search term '失智预防')

| Variable  Median (IQR) | Overall (n=130) | Governmental organizations | Commercial organizations | Medical institutions | Media or social organizations | Individuals | *p* value |
| --- | --- | --- | --- | --- | --- | --- | --- |
| GQS ^a^ | 3.0 (1.0) | 3.0 (1.0) | 3.0 (1.0) | 3.0 (0.0) | 3.0 (2.0) | 3.0 (2.0) | 0.878 |
| PEMAT-P | | | | | | | |
| Understandability (%) | 80.5 (13.0) | 88.0 (8.0) | 80.0 (13.0) | 81.5 (13.0) | 80.0 (23.0) | 75.0 (11.0) | 0.002* |
| Actionability (%) | 80.0 (23.0) | 83.0 (20.0) | 67.0 (21.0) | 80.0 (23.0) | 80.0 (17.0) | 67.0 (20.0) | 0.004* |
| Content completeness ^a^ | 4.5 (3.0) | 5.0 (3.0) | 4.0 (3.0) | 5.0 (2.0) | 5.0 (1.0) | 4.5 (2.0) | 0.468 |

Note: ^a^ Kruskal-Wallis H test comparing differences among the five publisher categories , **p*<0.05, ***p*<0.001

**Supplementary Table 2:**Correlation of PEMAT-P score, Content completeness, number of views and number of likes. (Results from searches conducted using the search term '失智预防')

| Variable | GQS | | PEMAT-P | | | |
| --- | --- | --- | --- | --- | --- | --- |
|  |  |  | Understandability | | Actionability | |
|  | *r* value | *p* value | *r* value | *p* value | *r* value | *p* value |
| Content completeness ^a^ | 0.783 | <0.001** | 0.162 | 0.66 | 0.286 | <0.001* |
| Number of views ^a^ | 0.061 | 0.488 | 0.113 | 0.200 | 0.130 | 0.141 |
| Number of Likes ^a^ | 0.076 | 0.390 | -0.007 | 0.936 | -0.001 | 0.994 |
| Advertising ^a^ | -0.419 | <0.001* | -0.165 | 0.061 | -0.223 | 0.008* |

Note: ^a^ spearman correlation analysis, **p*<0.05, ***p*<0.001

**Supplementary Table 3:**The difference analysis of different sources in GQS scores, PEMAT-P scores and content completeness.(Results from re-searches conducted using '痴呆预防' (dementia prevention) on August 28, 2025)

| Variable  Median (IQR) | Overall (n=152) | Governmental organizations | Commercial organizations | Medical institutions | Media or social organizations | Individuals | *p* value |
| --- | --- | --- | --- | --- | --- | --- | --- |
| GQS ^a^ | 3.0 (1.0) | 3.0 (1.0) | 3.0 (2.0) | 3.0 (1.0) | 3.0 (1.0) | 3.0 (1.0) | 0.198 |
| PEMAT-P | | | | | | | |
| Understandability (%) | 87.5 (13.0) | 90.5 (6.0) | 87.5 (8.0) | 87.5 (13.0) | 87.5 (21.0) | 78.6(19.0) | <0.001* |
| Actionability (%) | 80.0 (13.0) | 83.0 (20.0) | 80.0 (18.0) | 80.0 (0.0) | 80.0 (20.0) | 67.0 (20.0) | <0.001* |
| Content completeness ^a^ | 4.5 (3.0) | 5.0 (2.0) | 4.0 (4.0) | 5.0 (2.0) | 4.0 (3.0) | 4.0 (2.0) | 0.520 |

Note: ^a^ Kruskal-Wallis H test comparing differences among the five publisher categories , **p*<0.05, ***p*<0.001

**Supplementary Table 4:**Correlation of PEMAT-P score, Content completeness, number of views and number of likes(Results from re-searches conducted using '痴呆预防' (dementia prevention) on August 28, 2025)

| Variable | GQS | | PEMAT-P | | | |
| --- | --- | --- | --- | --- | --- | --- |
|  |  |  | Understandability | | Actionability | |
|  | *r* value | *p* value | *r* value | *p* value | *r* value | *p* value |
| Content completeness ^a^ | 0.669 | <0.001** | 0.002 | 0.984 | 0.228 | 0.005* |
| Number of views ^a^ | 0.105 | 0.198 | 0.022 | 0.790 | 0.135 | 0.096 |
| Number of Likes ^a^ | 0.053 | 0.516 | -0.057 | 0.484 | 0.101 | 0.217 |
| Advertising ^a^ | -0.369 | <0.001* | -0.064 | 0.433 | -0.164 | 0.043* |

Note: ^a^ spearman correlation analysis, **p*<0.05, ***p*<0.001

**Supplementary Table 5**: Global Quality Score (GQS) (Scoring ranges from 1 to 5).

| Score | Definition |
| --- | --- |
| 1 | Poor quality, poor flow of the video, most information missing, not at all useful for patients |
| 2 | Generally poor quality and poor flow, some information listed but many important topics missing, of very limited use to patients |
| 3 | Moderate quality, some important information is adequately discussed |
| 4 | Good quality good flow, most relevant information is covered, useful for patients |
| 5 | Excellent quality and flow, very useful for patients |

Note: The GQS evaluates Information quality using a five-point scoring system from poor (1) to excellent (5) quality.

**Supplementary Table** **6:** Patient Education Materials Assessment Tool for Print Materials (PEMAT-P) Scoring standard**.**

| Item | Description |
| --- | --- |
| Section 1: Understandability | |
| 1 | The material makes its purpose completely evident |
| 2 | The material does not include information or content that distracts from its purpose |
| 3 | The material uses common, everyday language |
| 5 | The material uses the active voice |
| 6 | Numbers appearing in the material are clear and easy to understand |
| 7 | The material does not expect the user to perform calculations |
| 8 | The material breaks or "chunks" information into short sections |
| 9 | The material's sections have informative headers |
| 10 | The material presents information in a logical sequence |
| 11 | The material provides a summary |
| 12 | The material uses visual cues (e.g., arrows, boxes, bullets, bold, larger font, highlighting) to draw attention to key points |
| 15 | The material uses visual aids whenever they could make content more easily understood (e.g., illustration of healthy portion size) |
| 16 | The material's visual aids reinforce rather than distract from the content |
| 17 | The material's visual aids have clear titles or captions |
| 18 | The material uses illustrations and photographs that are clear and uncluttered |
| 19 | The material uses simple tables with short and clear row and column headings |
| Section 2: Actionability | |
| 20 | The material clearly identifies at least one action the user can take |
| 21 | The material addresses the user directly when describing actions |
| 22 | The material breaks down any action into manageable, explicit steps |
| 23 | The material provides a tangible tool (e.g., menu planners, checklists) whenever it could help the user take action |
| 24 | The material provides simple instructions or examples of how to perform calculations |
| 25 | The material explains how to use the charts, graphs, tables, or diagrams to take actions |
| 26 | The material uses visual aids whenever they could make it easier to act on the instructions |

Note: Each item is rated as 'Yes' (1), 'No' (0), or 'Not Applicable (NA)'. A 'Yes' is given when 80-100% of the material meets the criterion.The final score is: (Total score / Maximum possible score) × 100.

**Supplementary Table 7** Codebook for Dementia Prevention Content Analysis on WeChat

| **Code** | **Definition** | **Examples** |
| --- | --- | --- |
| Education | Article provides education through teaching and identifying trigger factors that can lead to or worsen illness; understanding triggers and continuing prevention. | - Providing information about long-term    health education  - Helping patients recognize triggers during illness  - Learning to identify major triggers through symptoms |
| Healthy Lifestyle | Article provides health protection through proper diet, appropriate exercise, and good living habits; reducing disease risk factors. | - Dietary advice: balanced diet including multiple meals, whole grains, fruits  - Exercise advice: at least 150 minutes  of moderate exercise weekly  - Maintaining healthy body weight, avoiding obesity  - Quitting smoking, and limiting alcohol |
| Sensory Organ Protection | Article that protects organs such as eyes and ears and their health to prevent sensory disorders from affecting major aspects of life. | - Using assistive devices correctly  - Regular hearing screening for people with hearing impairments  - Regular hearing tests and vision checks  - Proper treatment for vision problems |
| Chronic Disease Control | Article about managing high blood pressure and other diseases that can lower the risk of chronic diseases. | - Adults should maintain blood pressure below 130mmHg or lower  - Treatment and control of high blood pressure  - Regular monitoring and treatment of high LDL cholesterol  - Regular monitoring of blood sugar and blood lipids |
| Social Interaction | Article about maintaining positive social relationships, reducing isolation and loneliness, and providing emotional support. | - Active participation in social activities  - Positive interactions with others  - Willingness to live with others  - Participation in group activities |
| Head and External Injury | Article about avoiding possible serious damage to external body parts; and reducing risks from daily activities. | - Wearing helmets when riding bicycles and motorcycles  - Preventing head injuries  - Stopping exercise when experiencing pain in body parts  - Being aware of risks during exercise |
| Mental Health Management | Article about managing pressure, focusing on emotions and mood, and other mental health issues to protect brain health. | - Regular physical and therapeutic exercise  - Seeking mental health support  - Maintaining a positive mental state  - Stress management techniques |
| Improving Air Environment | Article about reducing air pollution and harmful inhalation; reducing harmful substances that damage the lungs and nervous system, and protecting cognitive function. | - Reducing air pollution exposure  - Improving indoor air quality  - Special attention to air quality in high  pollution areas  - Personal protection measures |
| Traditional Chinese Medicine Prevention | Article about using traditional Chinese medicine theories and methods, through adjusting "qi and blood balance", and supplementing vital energy to prevent various diseases. | - Acupressure points  - Herbal therapy  - Chinese Medicine Exercises， Such as Tai Chi， and finger exercises. |
